# Fatal Liver Failure After Herpes-Simplex-Virus-2-Induced Acute Necrotizing Hepatitis: A Case Report

**DOI:** 10.1155/crhe/1414531

**Published:** 2025-07-23

**Authors:** Thomas Rösner, Julia Grenz, Marlene Schaumäker, Sven Cuntz, Norbert Blank, Alphonse Charbel, Christa Flechtenmacher, Patrick Michl, Jan Pfeiffenberger, Monica Boxberger, Isabelle Mohr

**Affiliations:** ^1^Department of Medical Oncology, National Center for Tumor Diseases (NCT), Heidelberg University Hospital, Medical Faculty Heidelberg, Heidelberg University, Heidelberg, Germany; ^2^Department of Gastroenterology and Hepatology, Heidelberg University Hospital, Medical Faculty Heidelberg, Heidelberg University, Heidelberg, Germany; ^3^Department of Hematology, Oncology and Rheumatology, Heidelberg University Hospital, Medical Faculty Heidelberg, Heidelberg University, Heidelberg, Germany; ^4^Institute of Pathology, Heidelberg University Hospital, Medical Faculty Heidelberg, Heidelberg University, Heidelberg, Germany

## Abstract

Herpes simplex virus (HSV) infections are common in the European population, typically presenting with mucocutaneous and anogenital manifestations. However, disseminated infections and organ involvement are rare, usually occurring in immunocompromised individuals, particularly after hematopoietic stem cell or solid organ transplantation. HSV1/2-induced hepatitis is infrequent but can result in acute liver failure (ALF) and increased mortality. We present a case of fulminant ALF caused by disseminated primary HSV2 infection in a fifty-year-old male with rheumatoid arthritis treated with the JAK-inhibitor upadacitinib for 3 months prior to presentation. Clinical examination revealed severe oropharyngeal mucositis and hepatic encephalopathy. Initial laboratory results showed bicytopenia, significantly elevated transaminases, bilirubin, inflammatory markers, and severe coagulopathy. Empirical treatment with an antimicrobial regimen, intravenous aciclovir, acetylcysteine, and plasmapheresis (PPH) was initiated. The patient was listed for urgent liver transplantation based on King's College criteria. Further investigations revealed a high viral load of HSV2 DNA in the blood, and transjugular liver biopsy confirmed extensive liver necrosis with positive HSV staining. Despite antiviral therapy, the HSV2 viral load remained high, indicating resistance, and the patient was deemed “nontransplantable” due to clinical deterioration with progressive hepatic coma, hemorrhagic–septic shock, multiorgan failure, and secondary bowel ischemia, ultimately leading to the patient's death from refractory shock. This is only the second documented case of fulminant ALF due to HSV2 hepatitis in a patient undergoing JAK inhibition, and the first involving upadacitinib. It highlights the importance of considering primary herpesvirus infection as a potential cause of ALF, particularly in immunocompromised patients, and underscores the need for early antiviral intervention to improve outcomes.

## 1. Introduction

Herpes simplex virus (HSV) types 1 and 2 have declining but still high seroprevalence in Europe and the United States, approximately 50%–60% and 12%–15%, respectively. Mucocutaneous and anogenital manifestations are common also in the immunocompetent [1–3]. However, in rare cases of disseminated primary infection or reactivation, particularly in immunocompromised hosts, there is a significant risk of severe organ involvement, including hepatitis, which can lead to acute liver failure (ALF). ALF is a rare but life-threatening condition characterized by acute liver injury, hepatic encephalopathy, and coagulopathy [4, 5].

HSV2 infection is an uncommon cause of hepatitis, and subsequent ALF is a rare complication, primarily reported in neonates, immunocompromised patients, and pregnant women, with mortality rates of 50%–90% [6–10]. The disease typically progresses rapidly, and the absence of specific symptoms can delay early diagnosis [6]. Early antiviral therapy is crucial and has been associated with improved survival. High-volume therapeutic plasma exchange or plasmapheresis (PPH), recommendation Grade 1A in the European Association of the Study of the Liver (EASL) guidelines on the management of ALF (fulminant), has shown efficacy in documented cases of HSV-induced ALF by removing immune and inflammatory mediators while simultaneously replenishing coagulation factors [10–13].

We present a case of fulminant, ultimately fatal ALF caused by disseminated primary HSV2 infection in a fifty-year-old male patient following upadacitinib therapy. We review the available literature and discuss clinical features for early recognition, diagnosis, and treatment options, including liver transplantation.

## 2. Case Presentation

A fifty-year-old male with a history of tobacco use (15 pack-years), bronchial asthma, and a 10-year history of rheumatoid arthritis presented to his family physician six weeks prior to hospitalization, reporting unilateral chest pain unresponsive to pain medication. He had no known allergies, worked as a truck driver, and lived alone. He mentioned a new romantic relationship lasting 4-5 weeks and denied recent travel, animal or chemical exposure, or the use of any additional medications, supplements, vitamins, or illicit drugs. His alcohol use was described as occasional (3-4 mixed beer drinks per week). The patient had been on oral prednisolone (10 mg daily) for several years, but the reason for the lack of tapering was unclear. Three months prior to hospitalization, he was switched to upadacitinib (Rinvoq), an oral JAK1 inhibitor. After initial improvement, the chest pain worsened again 1 week before hospitalization, prompting a chest x-ray and blood tests. These revealed subacute spontaneous triple rib fractures on the right side and elevated D-dimers. The patient was subsequently admitted to a local emergency room (ER), where a CT scan confirmed the rip fractures, and pulmonary embolism was ruled out. Incidentally, blood tests showed acute liver and kidney injury with coagulopathy and elevated inflammatory values, prompting transfer to our university hospital transplantation center for further evaluation and management. Upon admission to our ICU, the patient was afebrile, normotensive, but somnolent. Physical examination revealed severe hemorrhagic, erosive mucositis of the oral cavity, and right upper-quadrant abdominal tenderness, without hepatomegaly, guarding, or rebound tenderness. Skin exam showed ubiquitous, nonspecific scratch marks and two single whitish ulcers near the urethral meatus. Liver parenchyma appeared homogenously hyperechogenic on ultrasound, with intact macroscopic liver perfusion and no evidence of cholecystolithiasis or choledocholithiasis. No relevant pathological findings were noted on the electrocardiogram or transthoracic echocardiogram.

Initial laboratory results at admission were as follows: electrolytes and pH: within normal limits, serum ammonia: normal, leukocytes 2.1 /nL with severe lymphopenia, thrombocytes 45 /nL, c-reactive protein (CRP) 115 mg/L, procalcitonine (PCT) 6.6 ng/mL, creatinine 2.6 mg/dL, lactate dehydrogenase (LDH) 18,320 U/L, alanine aminotransferase (ALT) 10,095 U/L, aspartate aminotransferase (AST) 14,661 U/L, alkaline phosphatase (AP) 212 U/L, γ-glutamyl transferase (gGT) 112 U/L, total bilirubin 2.1 mg/dL, quick 33% (INR 2.0), partial thromboplastin time (pTT) 41 s, antithrombin-III-activity 60%, fibrinogen 1.0 g/L, and D-dimers > 35 mg/L. Further investigations revealed normal alpha1-antitrypsin levels, hypogammaglobulinemia with immunoglobulin-G-deficiency (3.0 g/L) especially, and compensatory hyper-alpha1/alpha2-globulinemia on serum electrophoresis. Immunofixation was negative for pathological findings. Serum ferritin was markedly elevated (72,000 μg/L). There were no other signs of iron or copper overload. A broad autoantibody screening was entirely negative.

The patient was promptly initiated on broad empirical antimicrobial therapy consisting of meropenem, vancomycin, and caspofungin, alongside an acetylcysteine regimen. Intravenous glucocorticoid therapy (methylprednisolone 1 mg/kg/day) was also started. Given the presence of extensive oral mucositis, an empirical antiviral therapy with aciclovir was promptly initiated after admission. Despite initial treatment, the patient's clinical condition progressively deteriorated over the first 48 h, leading to cardiocirculatory and respiratory failure, which required mechanical ventilation and escalating vasopressor support.

Following the King's College criteria for fulminant hepatic failure [14], the patient was urgently listed for liver transplantation after extensive multidisciplinary consultation and with consent from the patient's brother, who held power of attorney. Simultaneously, the patient underwent five cycles of high-volume plasma exchange (PPH). A hematological evaluation, including flow cytometry (FACS) and bone marrow biopsy, showed no signs of hematological malignancy in peripheral blood or bone marrow. Bone marrow analysis revealed reactive clonal rearrangements of the beta and gamma chains of the T-cell receptor but no evidence of macrophage activation syndrome (MAS). Viral serologies were negative for hepatitis B, hepatitis C, human immunodeficiency virus (HIV), hantavirus, and leptospira. Positive IgG with negative IgM was detected for hepatitis A, hepatitis E, Epstein–Barr virus (EBV), varicella zoster virus (VZV), and cytomegalovirus (CMV). Polymerase chain reaction (PCR) testing in the blood was negative for all viruses mentioned above. While anti-HSV-IgG was negative (due to the absence of a validated IgM assay at the time), PCR testing detected HSV-2 DNA in the patient's oral swab and bronchoalveolar lavage (BAL). Therefore, PCR testing from the peripheral blood was secondarily performed on Day 3.

**Figure 1:**
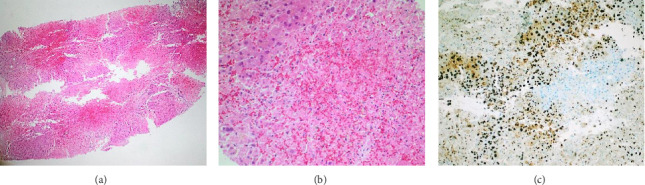
Transjugular needle biopsies of the liver showing hemorrhagically interspersed, confluent coagulative liver necrosis. (a) H&E stained slide, 100x magnification. (b) H&E stained slide, 400x magnification. (c) Immunoperoxidase stains for herpes simplex virus (HSV1/HSV2) with positive nuclear and cytoplasmic staining, indicating HSV-related hepatitis; 200x magnification.

On the fourth day after admission, results from a transjugular liver biopsy performed on Day 2 were available, revealing advanced acute hepatic damage with extensive hemorrhagic confluent coagulative liver necrosis in up to 70% of the biopsy material. Immunohistochemistry demonstrated clear nuclear reactivity of hepatocytes to antibodies against HSV1/HSV2. Representative images of the histopathological analysis are shown in Figure 1. In conjunction with the simultaneously available blood PCR results, which showed an extremely high HSV2 viral load of > 500.000.000 copies per mL, whereas HSV1 PCR was negative, diagnosis of disseminated primary HSV2 infection with necrotizing hepatitis was confirmed. Consequently, the patient was listed as inactive (“nontransplantable”) due to the severe, still uncontrolled viral infection and ongoing septic multiorgan failure being a contraindication against liver transplantation. Antiviral therapy and intensive supportive management were further continued.

**Figure 2:**
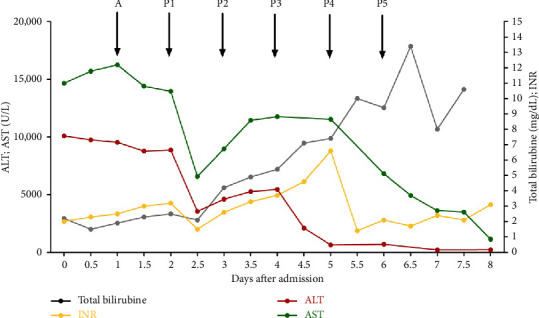
Trends of liver enzymes (AST and ALT), total bilirubin, and INR over the course of this patient's ICU stay. A. Initiation of intravenous aciclovir. P1–5. Initiation of plasmapheresis (cycles 1–5). AST: aspartate transaminase; ALT: alanine transaminase; INR: international normalized ratio.

Despite initial improvement in coagulation and inflammatory parameters following PPH, the patient developed progressive septic–hemorrhagic shock due to diffuse gastrointestinal bleeding, with no radiologically or endoscopically identifiable source of major hemorrhage. A cumulative mass transfusion was required. PPH was continued, and continuous veno-venous hemodialysis (CVVHD) was initiated for anuric renal failure (KDIGO 3), hypervolemia, and refractory hepatic encephalopathy. Methylprednisolone was switched to hydrocortisone (200 mg/day) according to local sepsis management protocols. Repeated laboratory tests and CT scans indicated likely secondary exudative pancreatitis, with serum lipase peaking at 2712 U/L, and rising levels of carboxyhemoglobin (CO-Hb) and methemoglobin (Met-Hb), likely due to inflammation-associated hemolysis and rhabdomyolysis. The levels of ALT, AST, INR, and bilirubin during the ICU stay are shown in Figure 2.

The patient's circulatory condition deteriorated, progressing to refractory shock and advancing insufficient tissue perfusion and lactate acidosis despite maximized vasopressor therapy. HSV2 viral load remained unchanged (> 500.000.000 copies per mL) in three follow-up measurements at 24 h, 96 h, and 120 h after initiation of high-dose aciclovir treatment. Given the patient's rapidly worsening condition without eligibility for a liver transplantation, a shared decision was made for palliative care. The patient died on Day 9 due to fulminant ALF and septic multiorgan failure.

## 3. Discussion

ALF of unknown origin presents a diagnostic challenge due to its rarity, diverse etiology, and the time-sensitive nature of therapy. While drug-induced ALF is the most common cause, viral hepatitis, ischemic hepatitis, and autoimmune disorders are of clinical relevance. According to the literature, up to 5%–10% of cases stay indeterminate despite extensive investigation [5].

HSV hepatitis is a rare cause of ALF, accounting for 0.8% of all cases and 2% of all viral hepatitis cases. It typically affects immunocompromised individuals and women during late pregnancy following a primary orogenital infection. However, up to 25% of cases occur in immunocompetent individuals. Most documented cases of HSV-associated ALF have involved disseminated primary HSV1 infections, but HSV2 has also been reported [6, 7, 9–12]. HSV viremia is characteristic of disseminated primary infection but rare in recurrent oral lesions [15]. Typical mucocutaneous or anogenital lesions are present in only 30%–50% of the documented cases of HSV hepatitis, and symptoms in general are described as mild, unspecific, and delayed as joint pain, fevers, and abdominal pain. Nevertheless, HSV hepatitis remains a critical differential diagnosis in fulminant ALF due to its high mortality of up to 90%, if untreated [6, 16, 17]. Common laboratory findings in HSV hepatitis include leukopenia, thrombocytopenia, renal failure, and disseminated intravascular coagulopathy. Hepatic encephalopathy is a late manifestation. Approximately 90% of patients exhibit an “anicteric hepatitis” profile, with a marked increase in transaminases up to 1000-fold, AST greater than ALT, and relatively low bilirubin [17, 18].

Histological confirmation, obtained through transcutaneous, transjugular, or laparoscopic liver biopsy, remains the gold standard. Biopsy findings include hemorrhagic necrosis, hepatocyte inflammation, enlarged nuclei with chromatin marginalization, and positive HSV immunostaining [19]. In our patient, the decision to perform a transjugular biopsy was made despite the increased risk. A transjugular approach was specifically chosen to minimize bleeding risk, as this method is considered safer in coagulopathic patients and is routinely used at our center in such critical scenarios. At the time of decision, the etiology of ALF was still unclear, as the biopsy was performed hours before the blood PCR results were obtained. Nevertheless, the primary purpose of the biopsy was not to confirm HSV hepatitis, but to evaluate the extent of acute hepatocellular injury and to identify any underlying chronic liver pathology and possible hepatic involvement of MAS, particularly in the light of the patient's ongoing urgent liver transplant listing.

This case represents the first documented instance of fatal HSV2-induced ALF in a patient receiving upadacitinib therapy. JAK inhibitors like upadacitinib are associated with an increased risk of viral infections, including herpesvirus reactivation, occurring in 3%–8% of patients depending on the dose and underlying disease [20, 21]. In 2020, Beck et al. reported the first case of fulminant HSV2-induced ALF in a patient on tofacitinib, who successfully underwent liver transplantation despite persistent pretransplant viremia [22]. Our patient's fatal outcome underscores the severity of this condition in the context of JAK1-mediated immunosuppression, and the fatality was reported to the drug manufacturer.

The gold standard for antiviral treatment in HSV hepatitis is aciclovir (5–10 mg/kg every 8 h, or adapted to kidney function) for 2–7 days, continued until clinical improvement, with a minimum total duration of 10 days. Early initiation is critical, as mortality rates decrease from 80%-90% to 50%–55% when treatment begins within 4 days of symptom onset [6].

Therefore, empiric aciclovir should be considered in patients with ALF or impending liver failure of unknown etiology, especially in those with immunosuppression or history of recurrent herpesvirus infections or typical lesions. Although rare, aciclovir resistance (reported in < 1% of HSV infections and up to 5% in immunocompromised patients) should be considered, with foscarnet as second-line treatment for when aciclovir resistance is suspected or confirmed [23]. In our case, aciclovir resistance was suspected due to persistent high viral loads (> 500.000.000 copies/mL) even 120 h after treatment initiation. It should be noted that the validated upper limit of quantification for the PCR assay in our institution is 5 × 10^8^ copies/mL, which implies that a declining viral load above this threshold could have remained undetected. Genotypic resistance testing was performed and revealed no relevant resistance mutation in the thymidine kinase. Additional phenotypic testing for resistance-mediating mutations of the viral polymerase (< 5% of resistant isolates) was not possible due to the lack of suitable material.

Systemic corticosteroids, such as methylprednisolone, are sometimes considered to reduce morbidity and mortality in viral hepatitis, although their impact remains inconclusive as a potential aggravation of the immunocompromised milieu in the patient is feared [7, 24]. Our patient received methylprednisolone early in the course of ALF, which was switched to hydrocortisone following the development of septic shock.

EASL guidelines recommend high-volume therapeutic plasma exchange or PPH as a cornerstone of supportive care in ALF, as it can help to remove inflammatory cytokines and replenish coagulation factors. PPH has also been shown to reduce viral load in disseminated HSV infections, although antiviral drugs like aciclovir are not significantly cleared by the procedure [10–12, 25, 26]. Interestingly, hemoadsorption may further enhance viral clearance, depending on the type of filter used [10].

Liver transplantation remains the definitive treatment for patients with fulminant ALF. While our patient was initially listed for transplantation, the diagnosis of HSV2-induced ALF, along with the presence of pretransplant viremia, ultimately precluded this option due to the severity of the underlying infection and refractory septic shock. Although liver transplantation can be successfully performed in HSV-induced ALF even in the presence of pretransplant viremia, particularly in children, it should be considered only in carefully selected adults with favorable prognostic factors, as documented morbidity and mortality are substantial [27–29]. Pretransplant antiviral therapy and lifelong reactivation prophylaxis are essential. As mentioned above, our patient disqualified for liver transplantation not solely because of the persistent viremia, but more importantly due to refractory septic multiorgan failure and clinically evident refractory coagulopathy despite PPH.

To conclude, HSV hepatitis is a rare but potentially fatal manifestation of disseminated HSV infection, which can occur in both immunocompromised and immunocompetent individuals. A detailed patient history, including former orogenital lesions, sexual activity, pregnancy, and immunosuppression, is essential. PCR testing for HSV should be included in the workup of every ALF, not only in patients harboring these risk factors, and empiric aciclovir therapy should be considered in patients with a high pretest probability, respectively. Liver biopsy remains the diagnostic gold standard, confirming the diagnosis by histological staining or bioptic PCR, even though the execution is associated with increased risk in this subset of patients and the therapeutic consequence may be questionable in some cases. Stress-dose glucocorticoids can be considered additionally due to a potential mortality benefit, although confirming evidence is still highly needed. PPH is effective in both viral clearance and supporting liver function in ALF patients, including those with disseminated HSV infection.

## Data Availability

The data that support the findings of this study are available from the corresponding author upon reasonable request.

## References

[1] Yousuf W., Ibrahim H., Harfouche M., Abu Hijleh F., Abu-Raddad L. (2020). Herpes simplex Virus Type 1 in Europe: Systematic Review, meta-analyses and meta-regressions. *BMJ Global Health*.

[2] Alareeki A., Osman A. M., Khandakji M. N., Looker K. J., Harfouche M., Abu-Raddad L. J. (2023). Epidemiology of Herpes simplex Virus Type 2 in Europe: Systematic Review, Meta-Analyses, and Meta-Regressions. *Lancet*.

[3] Bradley H., Markowitz L. E., Gibson T., McQuillan G. M. (2014). Seroprevalence of Herpes simplex Virus Types 1 and 2--United States, 1999–2010. *Journal of Infectious Diseases*.

[4] Bernal W., Wendon J. (2013). Acute Liver Failure. *New England Journal of Medicine*.

[5] Stravitz R. T., Lee W. M. (2019). Acute Liver Failure. *The Lancet*.

[6] Norvell J. P., Blei A. T., Jovanovic B. D., Levitsky J. (2007). Herpes simplex Virus Hepatitis: An Analysis of the Published Literature and Institutional Cases. *Liver Transplantation*.

[7] Ahmed A., Granillo A., Burns E. (2020). Herpes Simplex Virus-2 Hepatitis: A Case Report and Review of the Literature. *Case Reports in Medicine*.

[8] Boysen T., Clausen M. R. (2004). A Case of Acute Liver Failure Caused by Herpes Simplex Type 2. *Scandinavian Journal of Infectious Diseases*.

[9] Rimawi B. H., Meserve J., Rimawi R. H., Min Z., Gnann J. W. (2015). Disseminated Herpes Simplex Virus With Fulminant Hepatitis. *Case Reports in Hepatology*.

[10] Andermatt R., Bloemberg G. V., Ganter C. C. (2022). Elimination of Herpes simplex Virus-2 and Epstein-Barr Virus With Seraph 100 Microbind Affinity Blood Filter and Therapeutic Plasma Exchange: An Explorative Study in a Patient With Acute Liver Failure. *Critical Care Explorations*.

[11] Chávez S. M., Poniachik J. M., Urzua Á. M. (2021). Acute Liver Failure due to Herpes simplex Virus: Diagnostic Clues and Potential Role of Plasmapheresis: A Case Report. *Medicine (Baltimore)*.

[12] Holt E. W., Guy J., Gordon S. M. (2013). Acute Liver Failure Caused by Herpes simplex Virus in a Pregnant Patient: Is There a Potential Role for Therapeutic Plasma Exchange?. *Journal of Clinical Apheresis*.

[13] Wendon J., Cordoba J., Dhawan A. (2017). EASL Clinical Practical Guidelines on the Management of Acute (Fulminant) Liver Failure. *Journal of Hepatology*.

[14] O’Grady J. G., Alexander G. J., Hayllar K. M., Williams R. (1989). Early Indicators of Prognosis in Fulminant Hepatic Failure. *Gastroenterology*.

[15] Juhl D., Mosel C., Nawroth F. (2010). Detection of Herpes simplex Virus DNA in Plasma of Patients With Primary but Not With Recurrent Infection: Implications for Transfusion Medicine?. *Transfusion Medicine*.

[16] Ergle K., Caruso L., Burt M., Desai B., Patel R. (2015). Herpes Simplex Virus (HSV) in the Differential for Fulminant Hepatic Failure. *Case Reports in Clinical Medicine*.

[17] Poley R. A., Snowdon J. F., Howes D. W. (2011). Herpes simplex Virus Hepatitis in an Immunocompetent Adult: A Fatal Outcome due to Liver Failure. *Case Reports in Critical Care*.

[18] Peters D. J., Greene W. H., Ruggiero F., McGarrity T. J. (2000). Herpes Simplex-Induced Fulminant Hepatitis in Adults: A Call for Empiric Therapy. *Digestive Diseases and Sciences*.

[19] Natu A., Iuppa G., Packer C. D. (2017). Herpes simplex Virus Hepatitis: A Presentation of Multi-Institutional Cases to Promote Early Diagnosis and Management of the Disease. *Case Reports in Hepatology*.

[20] Danese S., Vermeire S., Zhou W. (2022). Upadacitinib as Induction and Maintenance Therapy for Moderately to Severely Active Ulcerative Colitis: Results From Three Phase 3, Multicentre, Double-Blind, Randomised Trials. *The Lancet*.

[21] Fleischmann R., Pangan A. L., Song I. (2019). Upadacitinib Versus Placebo or Adalimumab in Patients With Rheumatoid Arthritis and an Inadequate Response to Methotrexate: Results of a Phase III, Double-Blind, Randomized Controlled Trial. *Arthritis & Rheumatology*.

[22] Beck J., Haggard H., Membreno F. (2020). The First Documented Case of Fulminant Liver Failure From Herpes Simplex Virus in an Immunocompromised Patient Taking to Facitinib. *GastroHep*.

[23] Levin M. J., Bacon T. H., Leary J. J. (2004). Resistance of Herpes simplex Virus Infections to Nucleoside Analogues in HIV-Infected Patients. *Clinical Infectious Diseases*.

[24] Kluger N., Boutboul D., Molinari E. (2007). Acute Hepatitis During Primary Herpes Simplex Type 2 Infection in a Patient With Systemic Lupus Erythematosus. *Annales de Dermatologie et de Vénéréologie*.

[25] Zeidan J. H., Casingal V., Hippen B. (2021). Donor-Derived Herpes simplex Virus Hepatitis in a Kidney Transplant Recipient and Review of the Literature. *Transplant Infectious Disease*.

[26] Chavanet P. Y., Bailly F., Mousson C. (1990). Acyclovir Pharmacokinetics in Plasmapheresis. *Journal of Clinical Apheresis*.

[27] Shingina A., Mukhtar N., Wakim-Fleming J. (2023). Acute Liver Failure Guidelines. *American Journal of Gastroenterology*.

[28] Moldovan B., Mentha G., Majno P. (2011). Demographics and Outcomes of Severe Herpes Simplex Virus Hepatitis: A Registry-Based Study. *Journal of Hepatology*.

[29] Levitsky J., Duddempudi A. T., Lakeman F. D. (2008). Detection and Diagnosis of Herpes Simplex Virus Infection in Adults With Acute Liver Failure. *Liver Transplantation*.

